# Volumetric modulated arc therapy for hippocampal-sparing prophylactic cranial irradiation: Planning comparison of Halcyon and C-arm accelerators

**DOI:** 10.3389/fonc.2023.993809

**Published:** 2023-03-07

**Authors:** Tao Sun, Xiutong Lin, Kuo Li, Qingtao Qiu, Jinghao Duan, Guifang Zhang, Yong Yin

**Affiliations:** Department of Radiation Physics, Shandong Cancer Hospital and Institute, Shandong First Medical University and Shandong Academy of Medical Sciences, Jinan, Shandong, China

**Keywords:** small cell lung cancer, prophylactic cranial irradiation, hippocampalsparing, volumetric modulated arc therapy, Halcyon, Trilogy, TrueBeam

## Abstract

**Background:**

The purpose of the study was to evaluate the dosimetry of the Halcyon in prophylactic cranial irradiation (PCI) with volumetric modulated arc therapy (VMAT) and hippocampal-sparing for small cell lung cancer (SCLC).

**Methods:**

Five VMAT plans were designed on CT images of 15 patients diagnosed with SCLC and received PCI. Three plans with two full arcs were generated on the Trilogy and the TrueBeam accelerators, and flattening filter (FF) and flattening filter free (FFF) modes were used on TrueBeam. Two Halcyon plans with two and three full arcs were generated, referred to as H-2A and H-3A, respectively. The prescription dose was 25 Gy in 2.5-Gy fractions. The dose limit for hippocampus were D_100_ ≤ 9Gy and D_max_ ≤ 16Gy. The Wilcoxon matched-paired signed-rank test was used to evaluate the significance of the observed differences between the five plans.

**Results:**

H-2A plans significantly increased the D_2_ of PTV, and H-3A plans showed comparable or even better target dosimetry (better conformity) compared to the three plans on C-arm accelerators. Compared to T and TB plans, the two Halcyon plans significantly reduced the D_100_ and mean doses of bilateral hippocampus, the mean doses of eyeballs, and the maximum doses of lenses. D_100_ of hippocampus was reduced in TrueBeam plans comparing to Trilogy plans. The FFF plans on TrueBeam also represented advantages in D_mean_ and D_100_ of hippocampas, D_mean_ and D_max_ of eyeballs, and the D_max_ of lenses compared to FF plans. Halcyon plans and TrueBeam plans with FFF mode increased the MUs compared to FF plans. Comparing to H-2A, the H-3A plans exhibited additional dosimetric advantages, including D_2_, CI and HI of PTV, as well as the maximum and mean doses of hippocampus and eyeballs, and the maximum doses of optic nerves and brainstem. The two Halcyon plans significantly reduced the delivery time and showed the higher gamma passing rate than the three plans of C-arm accelerators.

**Conclusions:**

Compared with the C-arm accelerators, the dose of hippocampus and the delivery times on Halcyon are relatively significantly reduced for hippocampal-sparing PCI. Three arcs are recommended for VMAT plans with the Halcyon in hippocampal-sparing PCI.

## Introduction

Small cell lung cancer (SCLC) accounts for 15% of all lung cancers (1). SCLC evolves rapidly and has a tendency to metastasize early. Brain metastasis is one of the main failure modes after limited-stage SCLC treatment. The incidence of brain metastasis can reach 50% to 80% in patients who survive for more than two years (2).

Standard treatment for limited-stage SCLC includes chemotherapy and chest irradiation, followed by prophylactic cranial irradiation (PCI). The use of PCI could improve the overall survival and reduce the incidence of brain metastasis of patients with SCLC (3, 4). With the improvement of treatment technique and the standardization of treatment methods, the survival of SCLC patients has been prolonged, and the damage of neurocognitive function (NCF) caused by PCI has attracted more and more attention. Previous studies have shown that hippocampal irradiation is significantly correlated with NCF damage (5–7). However, some scholars have put forward a different view. The randomized phase 3 trial of NCT01780675 did not find that the probability of cognitive decline in SCLC patients who received hippocampal-sparing PCI (HS-PCI) was lower than that of traditional PCI (8). With the development of radiation technique, it is possible to protect the hippocampus in the design of treatment plan (7). Studies (9–11) have shown that volumetric modulated arc therapy (VMAT) has a significant dosimetric advantage over intensity modulated radiation therapy (IMRT) in hippocampal-sparing whole brain radiotherapy (HS-WBRT).

The Halcyon linear accelerator (LINAC) is the latest accelerator from Varian company, which was fitted with a single 6-MV flattening filter free (FFF) X-ray. Unlike conventional C-arm accelerators, the Halcyon machine has no jaw and is equipped with a double-layer multi-leaf collimator (MLC). The maximum gantry rotation speed of Halcyon is 4 times that of the C-arm LINAC, and the maximum leaf speed is 5 cm/s. Because of the faster gantry rotation and leaf movement speed, the treatment time of Halcyon can be relatively shortened. It was proved that the plan quality of Halcyon was maintained while the delivery times were reduced as compared with traditional C‐arm LINACs in the treatment of multisite tumors (12–14). Fewer delivery time bring out the benefits of less intra-fraction motion and more efficient patient throughput. Previous studies about HS-WBRT were based on conventional C-arm accelerators with jaw. Few studies have reported the application of VMAT plans on the Halcyon for SCLC patients with HS-PCI.

This study was based on the Varian Trilogy, TrueBeam and Halcyon accelerators. The HS-PCI VMAT plans on the three accelerators were designed for SCLC cases. The purpose of this study is to analyze the plan quality and delivery efficiency by comparing the dosimetric differences between the three accelerators to guide the clinical application in HS-PCI for SCLC cases.

## Materials and methods

### Patient selection and volume delineation

From October 2020 to June 2021, CT image datasets of 15 patients diagnosed with limited-stage SCLC and received PCI at Shandong Cancer Hospital were selected. **Table 1** shows the patient characteristics. All patients were simulated in the supine position with thermoplastic mask. The computed tomography (CT) and magnetic resonance (MR) simulation scans were performed in all patients. These images were fused and the target and organs at risk (OARs) were delineated in Varian Eclipse version 15.5 treatment planning system (Varian Medical Systems, Palo Alto, CA, USA).

**Table 1:** patient characteristics (n=15).

Characteristic	n
median age (range)	55(48-67)
Gender	
Male	10
Female	5
Stage of disease	
Limited	15
Extensive	0
Clinical AJCC stage	
IIA	4
IIB	6
IIIA	5

AJCC, American Joint Committee on Cancer.

The hippocampus was delineated on T1‐weighted MR imaging axial sequences following RTOG 0933 atlas definition (11). A hippocampal planning risk volume (PRV) was generated by the hippocampus expanded 5-mm margin. The planning target volume (PTV) was created by a 3‐mm extension of the whole brain and subtracting the hippocampal PRV. Other OARs were brainstem, bilateral optic nerves, eyeballs, and lenses and spinal cord.

### Treatment planning

Five VMAT plans were designed for each case. On the Trilogy, two 358° full coplanar arcs were designed. The collimator angles were 30° and 330°, respectively. The maximum dose rate was 600 MU/min in Trilogy plans (referred to as T). Two plans with the same beam angle as T plans were designed on TrueBeam accelerator, using FF and FFF modes respectively. In the two TrueBeam plans (referred to as TB and TB-FFF), jaw-tracking function was used. The maximum dose rates of TB and TB-FFF plans were 600 and 1400 MU/min, respectively. On the Halcyon version 1.0, two plans with two and three full arcs were designed, referred to as H-2A and H-3A, respectively. FFF mode was used with a maximum dose rate of 800 MU/min in the two Halcyon plans. Low-dose megavoltage cone beam computed tomography (MV CBCT) was selected for image guidance as Sun et al. (14) suggested for Halcyon plans.

6 MV X ray was used for all plans. The prescription dose was 25 Gy in 2.5-Gy fractions. All treatment plans were designed using the Eclipse version 15.5 treatment planning system (TPS) with an analytic anisotropic algorithm (AAA). During the optimization process, the volume of the lenses expanded by 3 mm was avoided to avert the direct entrance of the beam through the lenses. The dose limit for hippocampus followed RTOG 0933 protocol, which suggested D_100_ ≤ 9Gy and D_max_ ≤ 16Gy for hippocampus. Doses to 100% of the hippocampus in excess of 10 Gy and maximal hippocampal doses in excess of 17 Gy were considered unacceptable and require re-optimization of the plans. The optimization parameters of all plans were same, and the optimization goal was to minimize the dose to the OARs while ensuring the dose coverage of the PTV. In all plans, the maximum doses of lenses were less than 7Gy, the mean doses of eyeballs were less than 10 Gy. There were no dose limitations for the maximum doses to eyeball, brainstem, and spinal cord during the optimization process. The dose normalization was the prescription dose covered 95% volume of the PTV.

### Dosimetric evaluation

The dose statistics of the plans were based on dose-volume histogram (DVH) analysis. Because the dose normalization mode was the prescription dose covered 95% volume of the PTV, the coverages of PTV were 95% in all plans. For PTV, the dose of 2% and 98% volume (D_2_, D_98_) and the mean dose (D_mean_) were analyzed. The conformity index (CI), the homogeneity index (HI) and gradient index (GI) of the PTV were calculated using Equations (1), (2), (3), respectively. The three parameters were defined as following formulas:


(1) $$\text{CI}=\frac{TV_{PV}^{2}}{TV\times PV}$$

The TV_PV_ represents the volume of the PTV received the prescription dose, TV represents the volume of the PTV and PV represents the total volume received the prescription dose (15).


(2) $$\text{HI}=\frac{D_{2}-D_{98}}{D_{50}}$$

The D_2_, D_98_ and D_50_ represent the doses of 2%, 98% and 50% volumes of PTV, respectively (16).


(3) $$\text{GI}=\frac{V_{50}}{V_{100}}\;$$

The V_50_ and V_100_ represent the volumes of the 50% and 100% prescription dose line, respectively (17).

For hippocampus, dose received by 100% volume (D_100_), the mean dose (D_mean_), and the maximum dose (D_max_) were analyzed. For lenses, optic nerves, brainstem and spinal cord, the maximum doses (D_max_) were analyzed. For eyeballs, the mean dose (D_mean_), and the maximum dose (D_max_) were analyzed.

In order to assess the dose gradient between the hippocampus and the target, three ring structures were generated around the hippocampus (18). The first ring was a 5-mm three-dimensional ring around the hippocampus. The second and third rings were generated by applying a three-dimensional margin of 5 mm from the outer edges of the first and second rings respectively. The minimum dose (D_min_), the mean dose (D_mean_), and the maximum dose (D_max_) of the three rings were evaluated.

### Delivery verification

For all plans, the number of total monitor units (MUs) in each plan was calculated. The delivery time, from the first field beam on to the last field beam off, was recorded for each plan. The dose delivery efficiency was evaluated according to the total number of MUs and the delivery time. The dose quality assurance (QA) of the plans were performed using the electronic portal imaging device (EPID). The delivery accuracy was evaluated using the clinical gamma passing rate criteria of 3/2 (3%/2 mm) with a 10% lower dose exclusion threshold.

### Statistical analysis

All data were analyzed using the Statistical Package for Social Sciences v19.0 software (SPSS Inc., Chicago, IL, USA). Friedman’s two-way analysis of variance by ranks test for multiple samples was used to compared the H-2A and H-3A plans with the T, TB, and TB-FFF plans respectively. We also compared the differences between the H-2A and H-3A plans, and finally compared the T and TB plans, as well as the TB and TB-FFF plans. The Wilcoxon matched-paired signed-rank test was used to evaluate the significance of the observed differences for pairwise comparisons. The differences were considered statistically significant when *p*< 0.05.

## Results

### Volumes

The average volume of PTV was 1683.0 ± 160.9 cm^3^. The average volume of left hippocampus was 2.88 ± 1.61 cm^3^. The average volume of right hippocampus was 2.81 ± 1.45 cm^3^.

### Dose comparisons for Halcyon’s plans and the other two C-arm LINACs’ plans

**Figure 1:**
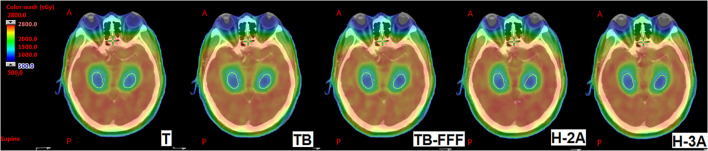
The dose distributions of the five plans for a representative patient. The images show T, TB, TB-FFF, H-2A and H-3A plans from left to right. The structures outlined in yellow are the bilateral hippocampus. A, Anterior; P, Posterior.

**Table 2** shows the dosimeric parameters of PTV and OARs for the five plans. For the mean dose and D_98_ of PTV, no significant differences were detected between the Halcyon’s plans and the other three C-arm LINACs’ plans. H-2A plans significantly increased the values of D_2_ of PTV compared with T and TB plans, and increased the values of HI of PTV compared to T plans. No significant differences were observed between H-3A plans and T, TB, TB-FFF plans in the two upper parameters and GI of PTV. TB-FFF plans showed the lower values in GI compared toH-2A plans, and no significant differences were observed between T, TB plans and H-2A plans. Compared withTB and TB-FFF plans, H-3A plans significantly increased the CI. **Figure 1** shows the dose distributions of the five plans for a representative patient.

**Table 2:** Dosimetric analysis for the five hippocampal-sparing plans.

	T	TB	TB-FFF	H-2A	H-3A	*p<*0.05
PTV						
D_mean_ (Gy)	26.08 ± 0.12	26.08 ± 0.12	26.09 ± 0.16	26.11 ± 0.08	26.03 ± 0.15	g
D_2_ (Gy)	27.02 ± 0.16	27.02 ± 0.17	27.08 ± 0.24	27.26 ± 0.13	26.99 ± 0.21	a,b,g
D_98_ (Gy)	23.72 ± 0.39	23.67 ± 0.40	23.64 ± 0.33	23.55 ± 0.27	23.46 ± 0.29	
CI	0.86 ± 0.01	0.86 ± 0.03	0.86 ± 0.03	0.87 ± 0.02	0.87 ± 0.02	e,f,g,
HI	0.13 ± 0.02	0.13 ± 0.02	0.13 ± 0.02	0.14 ± 0.01	0.14 ± 0.02	a,g
GI	1.61 ± 0.07	1.60 ± 0.07	1.58 ± 0.07	1.61 ± 0.07	1.60 ± 0.06	c,h,i
Hippo-L						
D_100_ (Gy)	9.01 ± 0.36	8.85 ± 0.49	8.63 ± 0.50	8.38 ± 0.44	8.17 ± 0.51	a,b,d,e,h
D_max_ (Gy)	14.86 ± 0.64	14.98 ± 0.54	14.90 ± 0.70	15.06 ± 0.69	14.69 ± 0.52	g
D_mean_ (Gy)	11.19 ± 0.38	11.21 ± 0.51	11.05 ± 0.54	10.99 ± 0.39	10.66 ± 0.40	d,e,g,i
Hippo-R						
D_100_ (Gy)	9.02 ± 0.44	8.82 ± 0.45	8.58 ± 0.47	8.35 ± 0.51	8.33 ± 0.40	a,b,d,e,h,i
D_max_ (Gy)	14.84 ± 0.80	14.99 ± 0.76	14.75 ± 0.86	14.96 ± 0.55	14.66 ± 0.56	g
D_mean_ (Gy)	11.22 ± 0.45	11.26 ± 0.50	10.97 ± 0.43	10.91 ± 0.44	10.68 ± 0.34	a,b,d,e,g,i
Eyeball-L						
D_mean_ (Gy)	7.98 ± 0.91	8.01 ± 1.00	7.66 ± 0.77	7.54 ± 0.72	7.14 ± 0.83	d,e,g,i
D_max_ (Gy)	18.40 ± 2.69	18.47 ± 2.44	18.07 ± 2.10	19.88 ± 1.89	18.65 ± 1.78	c,g,i
Eyeball-R						
D_mean_ (Gy)	8.10 ± 0.90	8.15 ± 0.99	7.85 ± 0.89	7.59 ± 0.71	7.17 ± 0.60	b,d,e,g,i
D_max_ (Gy)	18.01 ± 2.60	18.12 ± 2.84	17.54 ± 1.53	19.49 ± 2.12	18.09 ± 1.68	g.i
Lens-L						
D_max_ (Gy)	5.45 ± 0.62	5.52 ± 0.83	5.12 ± 0.70	4.48 ± 0.65	4.39 ± 0.79	a,b,d,e,i
Lens-R						
D_max_ (Gy)	5.46 ± 0.54	5.50 ± 0.82	5.02 ± 0.61	4.76 ± 0.73	4.44 ± 0.67	a,b,d,e,i
Opt-L						
D_max_ (Gy)	25.31 ± 0.80	25.43 ± 0.99	25.04 ± 1.06	25.76 ± 0.87	24.85 ± 0.64	g
Opt-R						
D_max_ (Gy)	25.69 ± 0.59	25.68 ± 0.51	25.49 ± 0.69	25.67 ± 0.89	25.05 ± 0.67	e,g
Brainstem						
D_max_ (Gy)	27.35 ± 0.22	27.40 ± 0.25	27.50 ± 0.37	28.11 ± 0.34	27.58 ± 0.30	a,b,c,g
Spinal cord						
D_max_ (Gy)	26.38 ± 1.87	26.33 ± 2.05	26.46 ± 1.66	26.77 ± 2.01	26.60 ± 1.91	a
Ring1						
D_max_ (Gy)	26.62 ± 0.51	26.56 ± 0.56	26.81 ± 0.66	24.53 ± 0.46	24.42 ± 0.64	a,b,c,d,e,f
D_min_ (Gy)	9.49 ± 0.37	9.35 ± 0.51	9.12 ± 0.44	8.91 ± 0.49	8.89 ± 0.56	a,d,e,i
D_mean_ (Gy)	17.33 ± 0.58	17.35 ± 0.52	17.29 ± 0.58	16.65 ± 0.36	16.49 ± 0.43	a,b,c,d,e,f,g
Ring2						
D_max_ (Gy)	27.61 ± 0.32	27.56 ± 0.42	27.81 ± 0.39	27.64 ± 0.33	27.36 ± 0.31	f,g,i
D_min_ (Gy)	13.41 ± 1.24	13.20 ± 1.32	13.00 ± 1.38	13.07 ± 1.10	13.39 ± 1.29	–
D_mean_ (Gy)	23.44 ± 0.48	23.41 ± 0.42	23.50 ± 0.40	22.95 ± 0.47	22.92 ± 0.52	a,b,c,d,e,f
Ring3						
D_max_ (Gy)	27.76 ± 0.22	27.79 ± 0.31	27.94 ± 0.35	28.33 ± 0.25	27.77 ± 0.22	a,b,g
D_min_ (Gy)	18.93 ± 1.82	18.74 ± 1.60	18.72 ± 2.07	19.38 ± 1.70	19.74 ± 1.84	d,e,f
D_mean_ (Gy)	25.65 ± 0.17	25.63 ± 0.18	25.69 ± 0.18	25.82 ± 0.21	25.72 ± 0.22	a,b,c,g,i

T, Trilogy plans; TB, TrueBeam plans; TB-FFF, TrueBeam plans with FFF mode; H-2A, Halcyon plans with two arcs; H-3A, Halcyon plans with three arcs; a, H-2A vs. T; b, H-2A vs. TB; c, H-2A vs. TB-FFF; d, H-3A vs. T; e, H-3A vs. TB; f, H-3A vs. TB-FFF; g, H-2A vs. H-3A; h, T vs. TB; i, TB vs. TB-FFF; Hippo-L, left hippocampus; Hippo-R, right hippocampus; Eyeball-L, left eyeball; Eyeball-R, right eyeball; Lens-L, left lens; Lens-R, right lens; Opt-L, left optic nerve; Opt-R, right optic nerve.

**Figure 2:**
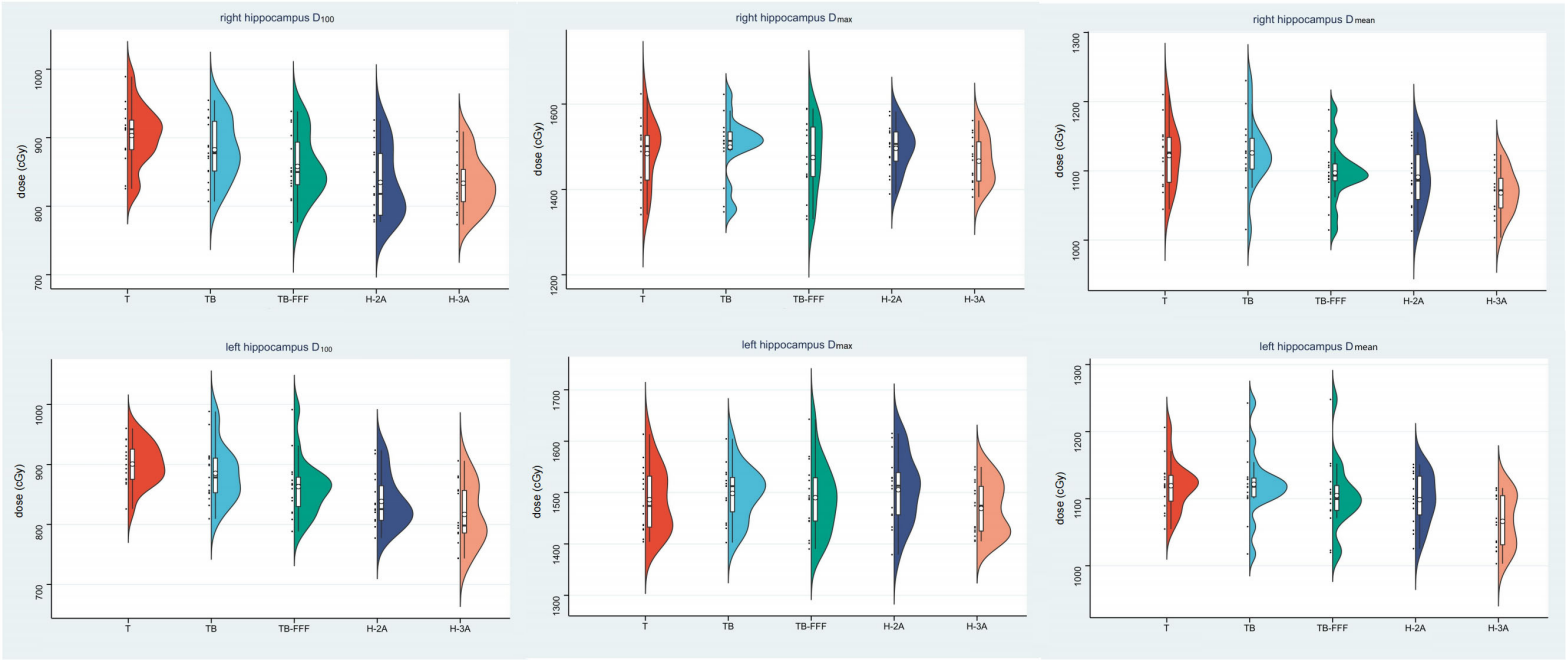
The half violin plot of D_mean_, D_max_ and D_100_ of the bilateral hippocampus for the five plans. The black horizontal line in the box shows the median value in each plan, and the upper and lower edges in the white box represent the upper and lower quartiles in the data set. The black points on the left half represent the specific values of this indicator for each case and plan.

**Figure 3:**
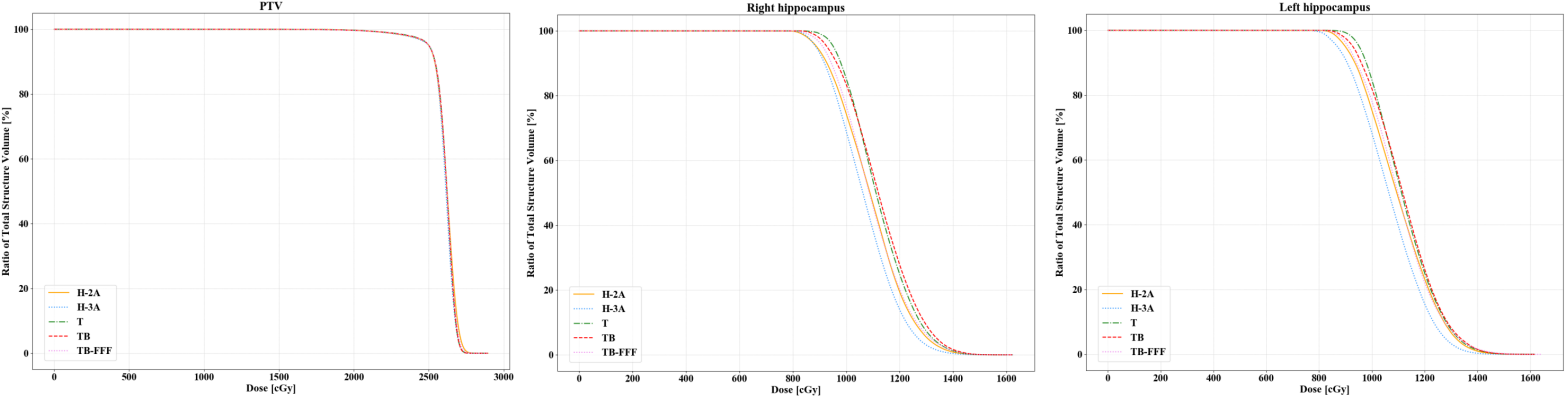
The mean dose-volume histograms of PTV and hippocampus for the five plans.

For the bilateral hippocampus, the two Halcyon plans significantly reduced the D_100_ compared to Trilogy and TrueBeam plans with FF mode. No significant differences were detected between the two Halcyon plans with TB-FFF plans in D_100_ of the bilateral hippocampus. For the mean dose of left hippocampus, H-3A plans showed the lower values compared to T and TB plans, and no significant differences were observed when comparing H-2A plans with T, TB and TB-FFF plans respectively. For the mean dose of right hippocampus, the two Halcyon plans showed lower values compared to Trilogy and TrueBeam plans with FF mode, and no significant differences were detected between the two Halcyon plans with TB-FFF plans. For the maximum dose of the bilateral hippocampus, there were no statistical differences between the two Halcyon plans and the other three plans. **Figure 2** shows the half violin plot of D_mean_, D_max_ and D_100_ of the bilateral hippocampus for the five plans. **Figure 3** and **Supplementary Figures 1-4** show the mean dose-volume histograms of PTV and OARs for the five plans.

For the mean doses of left and right eyeballs, the H-3A plans showed the lower values compared with T and TB plans. H-2A plans increased the maximum dose of left eyeball compared to TB-FFF plans. No significant differences were observed between the two Halcyon plans and the three plans of C‐arm LINACs on the maximum dose of right eyeballs. Compared to T and TB plans, the two Halcyon plans significantly reduced the maximum dose of bilateral lenses (*p*<0.01). For the maximum dose of left optic nerve, there were no statistical differences when comparing the two Halcyon plans with T, TB and TB-FFF plans. For the right optic nerve, the H-3A plans significantly reduced the maximum dose compared to the TB plans, and there were no statistical differences in this aspect between the H-2A plans and the three plans of C-arm LINACs. H-2A plans increased the maximum doses of brainstem compared to T, TB and TB-FFF plans, and increased the maximum doses of spinal cord compared to T plans. No statistical differences were observed between H-3A plans and the three plans of C-arm LINACs on the maximum doses of brainstem and spinal cord.

Compared to the three C-arm LINACs’ plans, the two Halcyon plans reduced the maximum dose of ring1 and the mean dose of ring1 and ring2, and H-2A plans increased the mean dose of ring3, but the increase in H-2A plans was very slight.

### Dose comparisons for the two Halcyon plans

Two Halcyon plans with different arcs were compared. For the PTV, H-3A plans showed the lower D_mean_ and D_2_ compared to H-2A plans. No significant differences were observed in D_98_ among the two plans. H-3A plans improved the CI and HI compared with H-2A plans.

H-3A plans significantly reduced the maximum and mean dose of bilateral hippocampus (*p*<0.01). In the D_mean_ and D_max_ of bilateral eyeballs, and D_max_ of bilateral optic nerves and brainstem, significant decreases were achieved with the three-arc Halcyon plans compared to the two-arc plans. No statistically significant differences were observed in the maximum dose of bilateral lenses between the H-2A and H-3A plans.

For rings, H-3A plans showed the lower values in the Dmean of ring1, the Dmax of ring2, and the Dmax and Dmean of ring3 compared to H-2A plans.

### Dose comparisons for the Trilogy and the TrueBeam plans

In TrueBeam plans, jaw-tracking was used. Trilogy accelerator does not have this function. For the PTV, when comparing Trilogy and TrueBeam plans, only the GI showed statistically different, and no significant differences were observed in D_98_, D_2_, D_mean_, CI and HI. The TrueBeam plans showed the lower GI compared to the Trilogy plans.

For the OARs, only the D_100_ of the bilateral hippocampus showed statistically different between the two plans, with the TrueBeam plans showing the lower values. There were no statistical differences in other analysis parameters of OARs and rings between the two plans.

### Dose comparisons for the two TrueBeam plans

Two TrueBeam plans with FF and FFF modes were compared. For PTV, only the GI showed statistically different and lower in FFF plans comparing to the FF plans. There were no statistical differences in other parameters including D_mean_, D_2_, D_98_, CI and HI between the two plans.

FFF plans reduced the D_mean_ of left hippocampus and the D_100_ and D_mean_ of right hippocampus compared to FF plans. For the D_100_ of left hippocampus, FFF plans showed the lower average values, but no statistical significance was found. In terms of D_mean_ and D_max_ of bilateral eyeballs, and the D_max_ of bilateral lenses, FFF plans showed the lower values significantly. No significant differences were observed in the maximum dose of bilateral optic nerves, brainstem, and spinal cord between the two plans. FFF plans reduced the D_min_ of ring1, and increased the D_max_ of ring2 and D_mean_ of ring3, but the differences were very small.

### Delivery efficiency

**Table 3** shows the numbers of MUs and the delivery time of the five plans. TB-FFF and two Halcyon plans increased the numbers of MUs significantly compared to T and TB plans (*p*=0.001). For the numbers of MUs, pairwise comparisons between TB-FFF, H-2A and H-3A plans showed no statistical difference, and the comparison between T and TB plans showed the same results. Compared to the three plans of C-arm accelerators, the two Halcyon plans significantly reduced the delivery time (*p*=0.001). H-3A plans significantly increased the delivery time compared to H-2A plans (*p*=0.001).

**Table 3:** Delivery efficiency and gamma evaluation passing rates of the five plans.

	T	TB	TB-FFF	H-2A	H-3A	*p*<0.05
**MUs**	760.3 ± 96.9	759.7 ± 120.8	900.1 ± 115.6	901.0 ± 105.8	912.0 ± 109.0	a,b,d,e,i
**Delivery time (min)**	3.2 ± 0.2	3.1 ± 0.2	3.1 ± 0.2	1.5 ± 0.1	2.1 ± 0.1	a,b,c,d,e,f,g
**Passing rate (%)**	96.0 ± 0.9	96.3 ± 1.3	96.2 ± 1.2	97.5 ± 1.1	97.4 ± 1.0	a,b,c,d,e,f

T, Trilogy plans; TB, TrueBeam plans; TB-FFF, TrueBeam plans with FFF mode; H-2A, Halcyon plans with two arcs; H-3A, Halcyon plans with three arcs; a, H-2A vs. T; b, H-2A vs. TB; c, H-2A vs. TB-FFF; d, H-3A vs. T; e, H-3A vs. TB; f, H-3A vs. TB-FFF; g, H-2A vs. H-3A; h, T vs. TB; i, TB vs. TB-FFF.

### QA

**Table 3** shows the gamma passing rates of the five plans. The gamma passing rates of all plans met the standard of our institution (≥95%). **Supplementary Table 1** shows the specific QA results for the 15 patients. The two Halcyon plans showed the higher gamma passing rate than the three C-arm LINACs’ plans. No statistical differences were observed between the two Halcyon plans (97.5 ± 1.1% vs. 97.4 ± 1.0%).

## Discussion

Efficient workflows during radiotherapy provide the benefits of higher clinical throughput and improving patient comfort, as well as reducing the cost per treatment by reducing machine and staff time (19). It was proved that Halcyon was more efficient than conventional C-arm accelerators, and the Halcyon plans demonstrated comparable planning quality to C-arm accelerators in tumors at multiple sites (12–14). In this study, Halcyon also showed similar planning quality to the C-arm accelerators in hippocampal-sparing PCI for SCLC cases.

For analyzed parameters of PTV, H-2A plans increased D_2_ compared to Trilogy and TrueBeam plans with FF mode, and showed similar CI and HI compared to the three C-arm LINACs’ plans. H-3A plans increased conformity and showed similar dosimetry in other parameters of PTV compared to the three C-arm LINACs’ plans. Michiels et al. (13) compared the plan quality of Halcyon and TrueBeam for VMAT of head-and-neck cancers, and they found that Halcyon plans showed a lower target dose homogeneity than TrueBeam plans. The results were similar to ours. In our study, the Halcyon plans with two arcs showed lower homogeneity and higher D_2_ compared to one or two of the three C-arm LINACs’ plans. The average increase for the D_2_ of PTV was about 0.2 Gy in H-2A plans compared to the plans of Trilogy and TrueBeam. The increment was small. The reason why D_2_ was relatively higher in H-2A plans may be that only the FFF beam was available on the Halcyon. In this study, H-2A plans showed similar HI and D_2_ compared to VB-FFF plans. Flattening filter is used to achieve uniform dose coverage. Bhushan et al. (20) found that VMAT plans with FFF mode increased D_2_ and HI compared to FF mode for the treatment of gastric tumors.The Halcyon plans with three arcs showed comparable or even better target dosimetry (better conformity) compared to the three plans of Trilogy and TrueBeam accelerators.

The application of PCI could reduce the incidence of brain metastases and prolong disease-free survival in SCLC patients, but PCI has an impact on the development of neurocognitive impairment. As long-term cancer survivors increase, specific measures need to be taken to limit these adverse effects. Preclinical and human studies have shown that bilateral or unilateral hippocampal radiation injury may be the key reason for the subsequent decline of NCF, especially in learning and memory (21, 22). Gondi et al. (11) found that a dose of more than 9 Gy in the 100% volume of hippocampus and a maximum hippocampal dose of more than 16 Gy were associated with memory functional impairment in WBRT of 30 Gy in 10 fractions. In this study, all five plans met this dose requirement. Halcyon plans reduced the D_100_ and mean doses of bilateral hippocampus compared to Trilogy and TrueBeam plans with FF mode. Compared with FFF plans on TrueBeam, Halcyon plans also showed lower D_100_ and mean dose of bilateral hippocampus, but no statistical significance was found. Trilogy and TrueBeam are equipped with the Varian Millennium-120 MLC. Li et al. (23) and Yao et al. (24) detected that the transmission ratio of Millennium-120 MLC was 1.4% and 1.5%, respectively, using different measurement methods. Halcyon is equipped with a dual-layer MLC with a very small dosimetric leaf gap (0.1 mm), which provides the advantages of ultra-low MLC leakage and transmission dose (less than 0.5%) and a reduction of penumbra (25). The measured single-layer MLC transmission ratio of Halcyon MLC was 0.42% (26) and the measured distal and proximal leaf transmission were 0.41% and 0.40%, respectively (27). The low transmission ratio and small penumbra of Halcyon may account for the lower D_100_ and mean dose of hippocampus in Halcyon plans. We found jaw tracking technique revealed some dosimetry advantage of reducing hippocampal dose. D_100_ of hippocampus was reduced in jaw-tracking plans. Kirby et al. (28) investigated the effect of collimator leakage for the neural stem cell (NSC) in whole-brain radiation therapy. The results showed that the use of jaw-tracking with the reduction of collimator leakage significantly reduced the dose of NSC. Their results are the same as ours. In our study, the FFF technique also represented advantages in some hippocampal dosimetric parameters (D_mean_ and D_100_). Ji et al. (29) revealed that VMAT plans with FFF beams could reduce the maximum, minimum, and mean doses to the hippocampus for WBRT. Our results are similar to theirs, except for the maximum dose in the hippocampus. In our study, the FFF plans showed the lower average maximum dose but no statistical difference was found. Previous study has confirmed that the MLC transmission can be further reduced by adopting FFF mode (30).

Compared with C-arm LINACs’ plans, Halcyon plans reduced the mean doses of eyeballs. International guideline in radiation therapy planning for nasopharyngeal carcinoma proposed the dose limit of eyeball is a mean dose of less than 35 Gy with a maximum acceptance criterion (MAC) of D_0.03cc ≤_ 50 Gy, and the limited dose of lens are D_0.03cc_ dose<6 Gy and MAC at D_0.03cc_ dose ≤15 Gy (31). In our study, eyeball doses in five plans all met this dose limit. There was no dose limitation on the maximum dose of eyeball during the planning optimization. H-2A plans increased the maximum doses of left eyeballs compared to TB-FFF plans. The increase of 1 Gy in the maximum dose of eyeball in Halcyon plans may not be clinically significant. The Halcyon plans significantly reduced the maximum doses of lenses compared to the other three plans. After an overexpose of the lens, germinative zone of the lens epithelium is damaged and the cells die, eventually leading to radiation-induced cataracts (32). In our study, all five plans met the recommended dose limits in the international guidelines above. The lower doses of eyeball and lens in Halcyon plans may be due to the low leakage and FFF configuration of the Halcyon accelerator. This is also seen in the FF versus FFF plans for TrueBeam, where both eyeball and lens doses are significantly lower in the FFF plan. This may be due to the characteristics of lower out-of-field dose in the FFF mode. Our results are identical with those of previous study from Ji et al. (29). H-3A plans showed the lowest average maximum dose of bilateral optic-nerves. The significant difference was found only in the right optic nerve between H-3A and TB plans. In our planning optimization process, the weight of dose constraint on the optic nerve was not large. In two cases, the maximum doses of the left optic nerve were higher in H-3A plans (about 100cGy higher than those in TB plans). This may be the reason why there was no statistical difference in the maximum dose of the left optic nerve between H-3A and TB plans.

In RTOG 0933, 98% of the target volume should receive at least 25 Gy to avoid cold spots in the brain, which may lead to an increase in local recurrence (LR). In WBRT or PCI with hippocampal-sparing, the region with the lowest dose is usually near the hippocampal avoidance region. Compared with WBRT alone, cold spots in this region may lead to an increase in LR. In SCLC, LR can increase by 4% (33). Therefore, special care needs to be taken in planning optimization to achieve a high dose gradient around the hippocampus. In this study, we analyzed the doses around the hippocampus. In an area of 5 mm around the hippocampus avoidance region (Ring 2), the mean doses of about 23Gy were achieved in all five plans, which was close to the prescribed dose of 25 Gy. Halcyon plans showed the lowest D_min_, D_max_ and D_mean_ of Ring1. Low doses in this region may increase the robustness of hippocampal-sparing.

Halcyon plans and TrueBeam plans with FFF mode increased the MUs compared to FF plans. This is mainly due to the characteristics of FFF beam, in which more MUs are required in a point far away from the central axis to achieve the same depth dose as the central point. The results are consistent with previous studies (29, 34, 35). Although Halcyon plans has increased MUs compared to FF plans, the delivery time was still significantly reduced with Halcyon compared to C-arm LINACs’ plans whether using FFF or FF in this study. Compared to the C‐arm LINACs, the speeds of Halcyon are up to 4 and 2 times faster during the imaging and dose delivery phases, respectively. Faster treatment can be achieved based on the higher gantry rotation speed and MLC leaf speed. The shorter treatment times can reduce intra-fraction motion, improve patient comfort and increase machine throughput.

When comparing Halcyon plans with three arcs and two arcs, we found that the three arcs plans exhibited additional dosimetric advantages, including D_2_, CI and HI of PTV, as well as the maximum and mean doses of hippocampus and eyeballs, and the maximum doses of optic nerves and brainstem. Previous studies have reported that the qualities of plans are improved as the number of arcs increases, i.e., from one arc to two arcs (36, 37) and from two arcs to four arcs (38). A study has suggested three arcs were implemented for VMAT plans of head and neck cancers on Halcyon (13). For VMAT plans of HS-PCI in SCLC patients, three arcs are also proposed for Halcyon according to present research. Although Halcyon plans with three arcs increased the delivery times compared with those with two arcs (2.1 ± 0.1 min vs. 1.5 ± 0.1 min), it significantly improved the dosimetry quality, and significantly reduced the delivery times compared with the other three C-arm LINACs’ plans (3.2 ± 0.2, 3.1 ± 0.2, and 3.1 ± 0.2 min for T, TB and TB-FFF plans, respectively).

The EPID of the Halcyon is the latest aSi-1200 with a resolution of 1280x1280. The distance from the source to the image board is fixed and the machine automatically scales the image board during the morning check every day. The EPIDs of the Trilogy and TrueBeam are aSi-1000, with a measurement resolution of 1024×768. During dose verification by EPID, the robot arm needs to be opened every time to lift the image board to the required position, which may lead to accuracy deviation. The above may be the reasons for the higher gamma pass rates in Halcyon plans comparing with Trilogy and TrueBeam plans in this study.

The six degree of freedom (6-DoF) couch could be used to correct rotation errors for Trilogy and TrueBeam LINACs. The couch of Halcyon only allows for correction of translational shifts. If a large rotation is observed on the initial image, repositioning and additional imaging may be required. This deficiency could significantly reduce the advantage of faster treatment with the Halcyon. Surface guided radiation therapy (SGRT) is particularly useful on Halcyon to correct rotation errors during patient positioning and monitor the patient’s motion during beam delivery (39). Flores-Martinez et al. (19) confirmed the use of SGRT on Halcyon reduces re-imaging during patient setup and improves accuracy of patient position by reducing residual rotation errors, and they also demonstrated that for cranial treatment using the Halcyon the most effective workflow is the one including SGRT for setup, KV CBCT for imaging and VMAT for delivery. In our study, Halcyon version 1.0 was adopted which equips only MV CBCT. However, we believe that combining SGRT and VMAT, positioning and treatment efficiency of the Halcyon can be significantly improved.

A limitation of this study was that the numbers of patient included in the study were less. Our study was based on the dosimetry of the plans rather than the clinical outcome. The reduction of hippocampal dose in Halcyon plans may not be clinically significant, but the high efficiency of treatment on the Halcyon machine will bring more benefits to patients. In the future, we may collect more cases for relevant prospective studies to study the clinical significance of hippocampal dose reduction.

## Conclusions

In hippocampal-sparing PCI for SCLC cases, Halcyon plans obtain similar target dosimetry, and all OARs could meet clinical requirements. Compared with the C-arm accelerators, the dose of hippocampus in Halcyon could be significantly reduced. Although the numbers of MUs are significantly increased compared to the plans with FF mode, the delivery times are relatively significantly reduced. Three arcs are recommended for VMAT plans with the Halcyon in hippocampal-sparing PCI.

## Data availability statement

The original contributions presented in the study are included in the article/**Supplementary Material**. Further inquiries can be directed to the corresponding author.

## Ethics statement

Written informed consent was not obtained from the individual(s) for the publication of any potentially identifiable images or data included in this article.

## Author contributions

TS and YY designed the study. GZ collected the CT data. TS, XL and KL designed the treatment plans. XL performed dose validation for all plans. QQ and JD analyzed the data. TS wrote the paper. All authors contributed to the article and approved the submitted version.
