# Supplementary material for: Volumetric modulated arc therapy for hippocampal-sparing prophylactic cranial irradiation: Planning comparison of Halcyon and C-arm accelerators
Source: Front Oncol. 2023 Mar 7;13:993809. doi: 10.3389/fonc.2023.993809 (PMC10028073; doi:10.3389/fonc.2023.993809)
Supplement: Supplementary file 1 [file DataSheet_1.pdf]

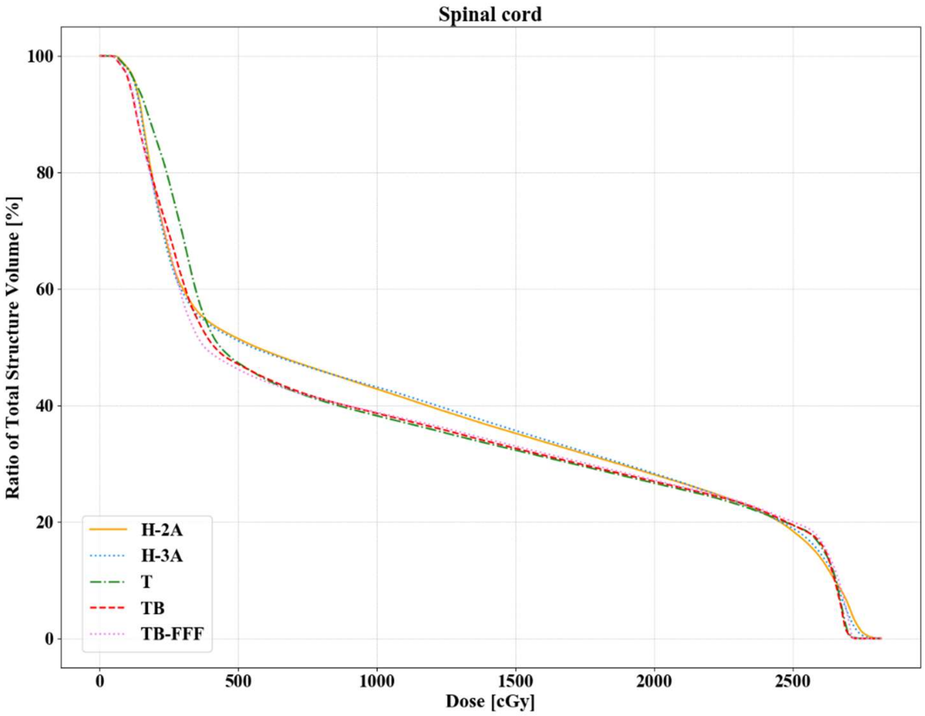

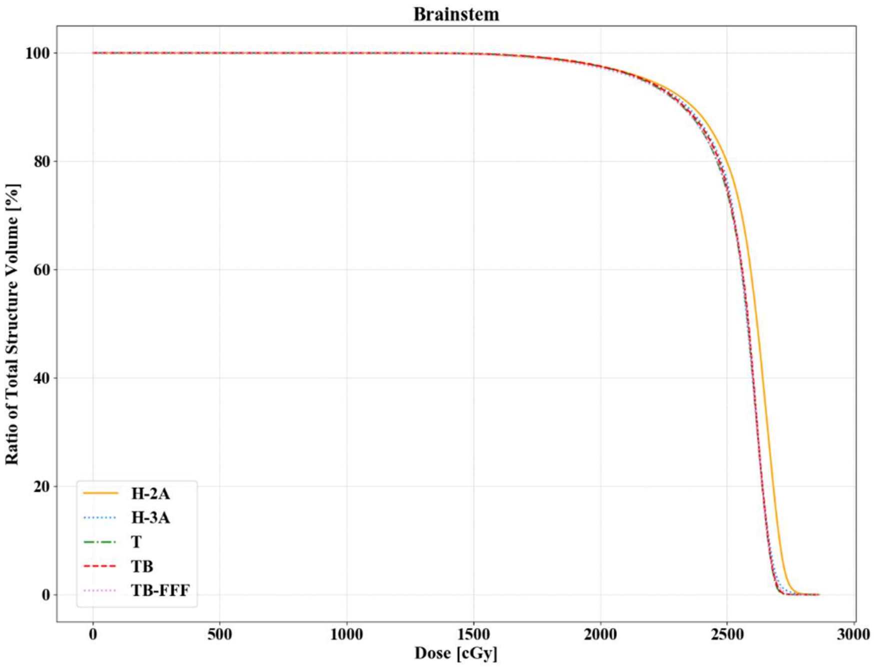

Supplementary Figure 1. The mean dose-volume histograms of spinal-cord and brainstem for the five plans.

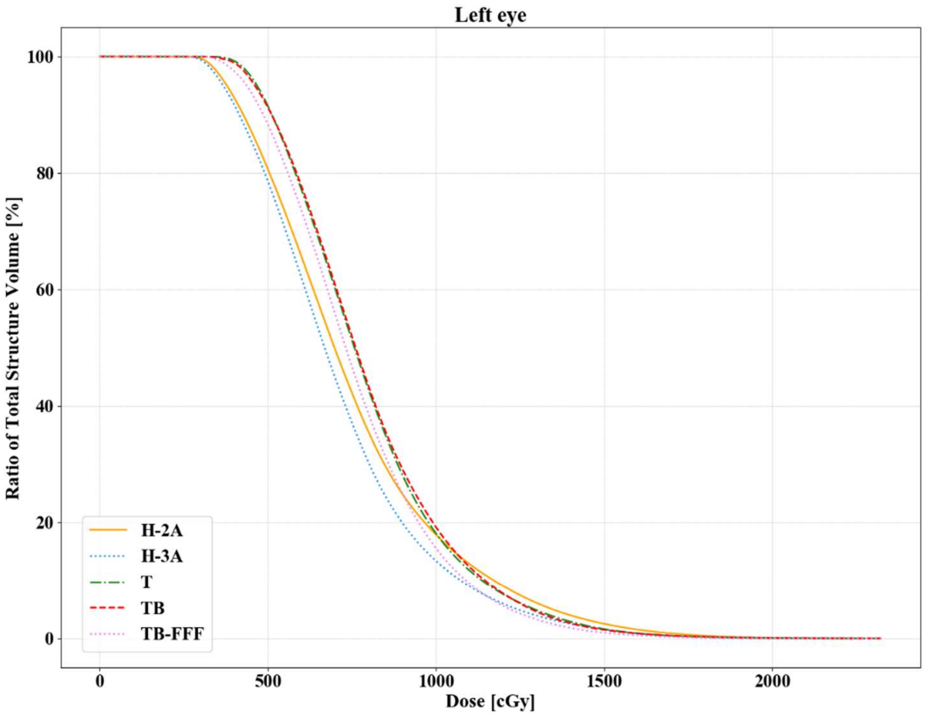

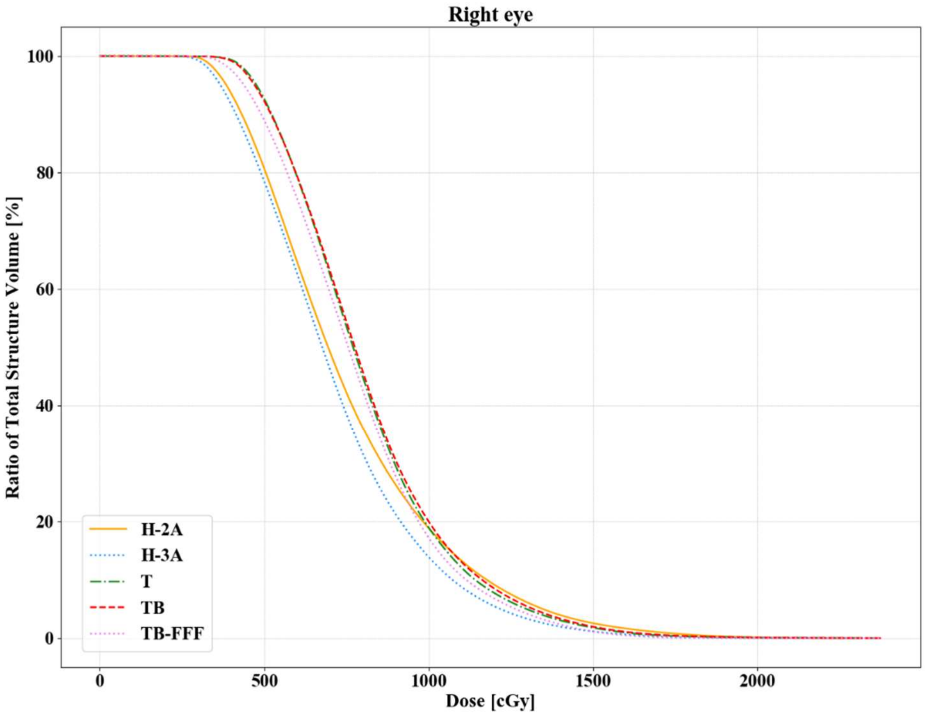

Supplementary Figure 2. The mean dose-volume histograms of left and right eyes for the five plans.

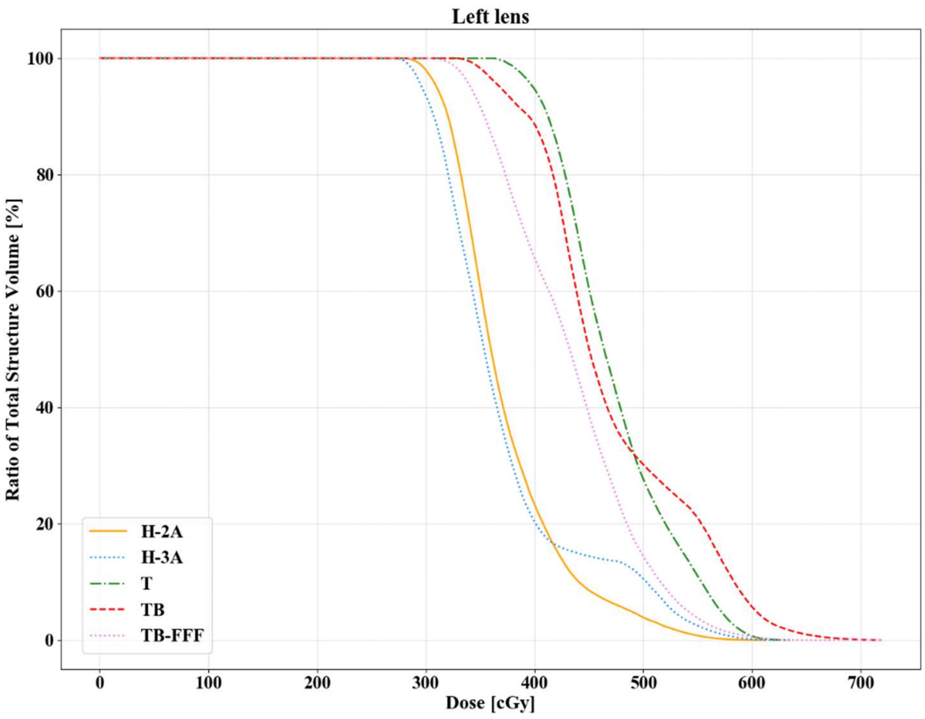

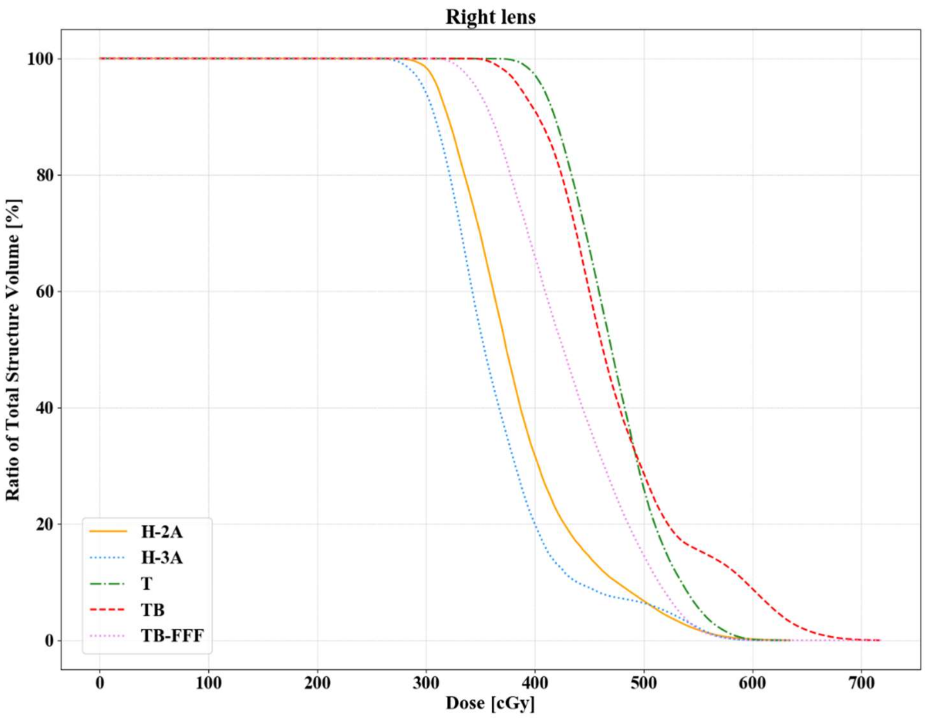

Supplementary Figure 3. The mean dose-volume histograms of left and right lenses for the five plans.

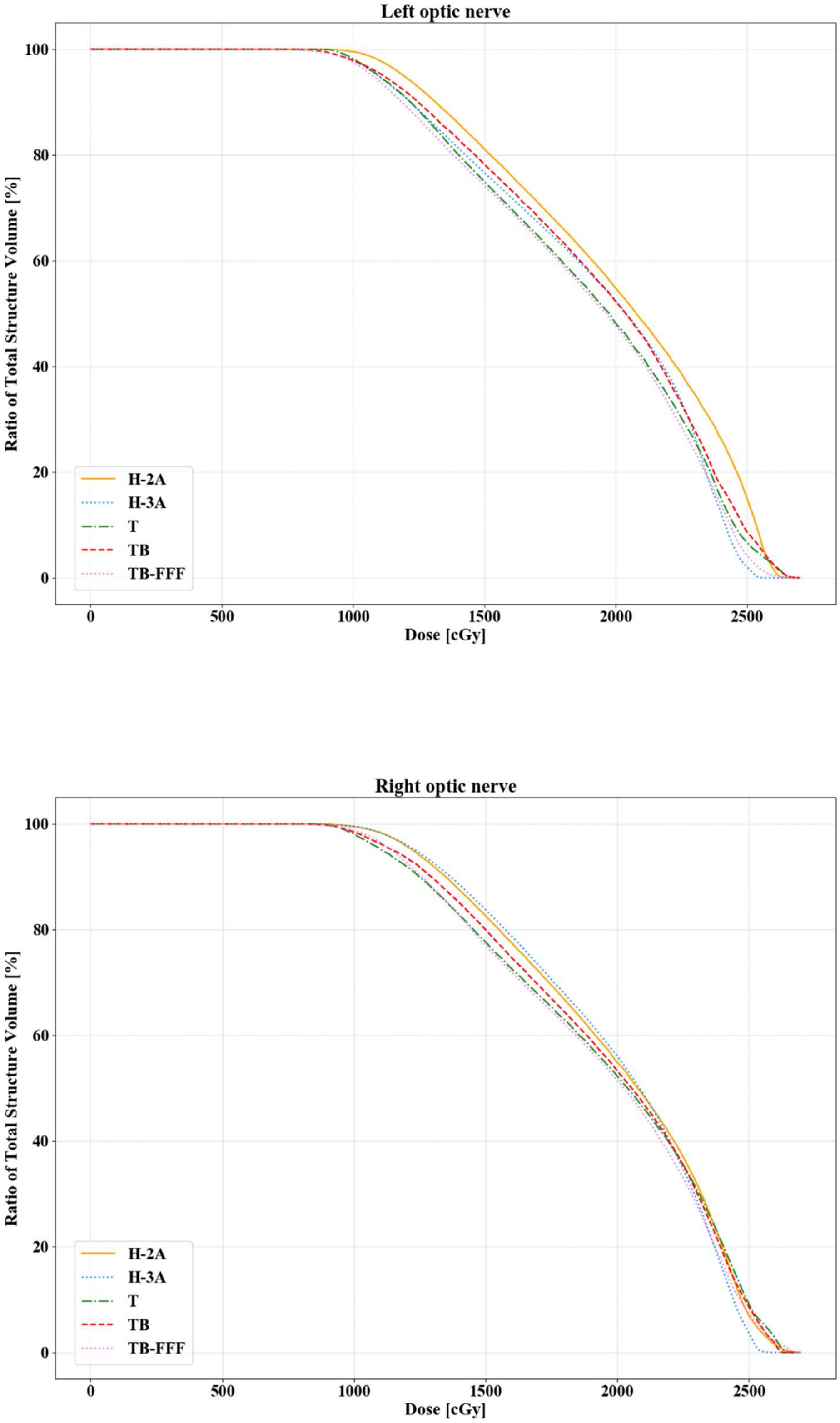

Supplementary Figure 4. The mean dose-volume histograms of left and right optic nerves for the five plans.

Supplementary Table 1. The gamma evaluation passing rates (%) of the five plans for the 15 patients

patient number	T (%)	TB (%)	TB-FFF (%)	H-2A (%)	H-3A (%)
1	95.1	95.2	95.1	95.1	95.2
2	96.7	97.8	96.9	98.7	98
3	95.2	95.3	95.3	95.8	96.2
4	95.9	96.1	96.1	98.1	97.9
5	95.3	95.4	95.4	96.8	96.8
6	95.8	95.7	95.8	97.4	97.7
7	95.9	95.9	95.8	97.9	97.7
8	95.5	95.6	95.5	97.4	97.4
9	95.7	95.6	95.7	97.4	97.7
10	98.3	98.9	98.8	98.8	98.9
11	96.1	96.1	96.4	98.2	98
12	95.4	95.5	95.4	97.1	96.8
13	96.1	96.5	96.4	98.8	98
14	95.3	95.3	95.4	96.6	96.5
15	97.2	98.9	98.8	98.8	98.8
